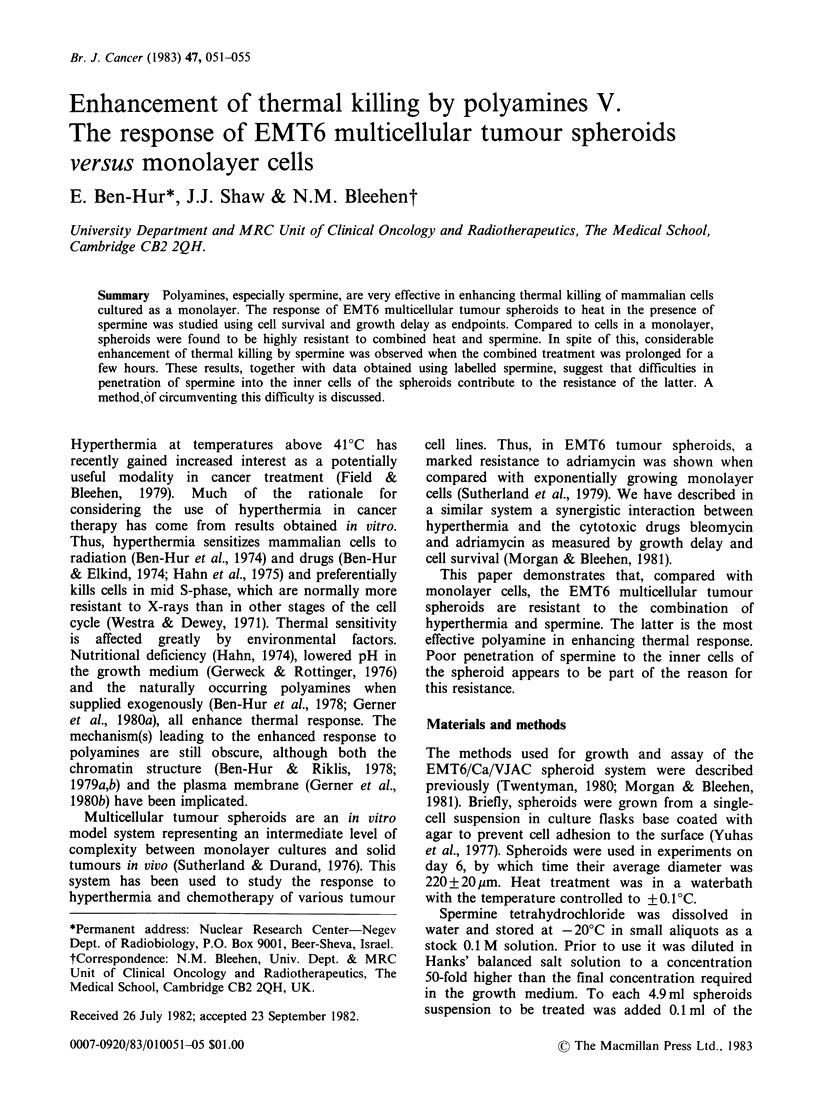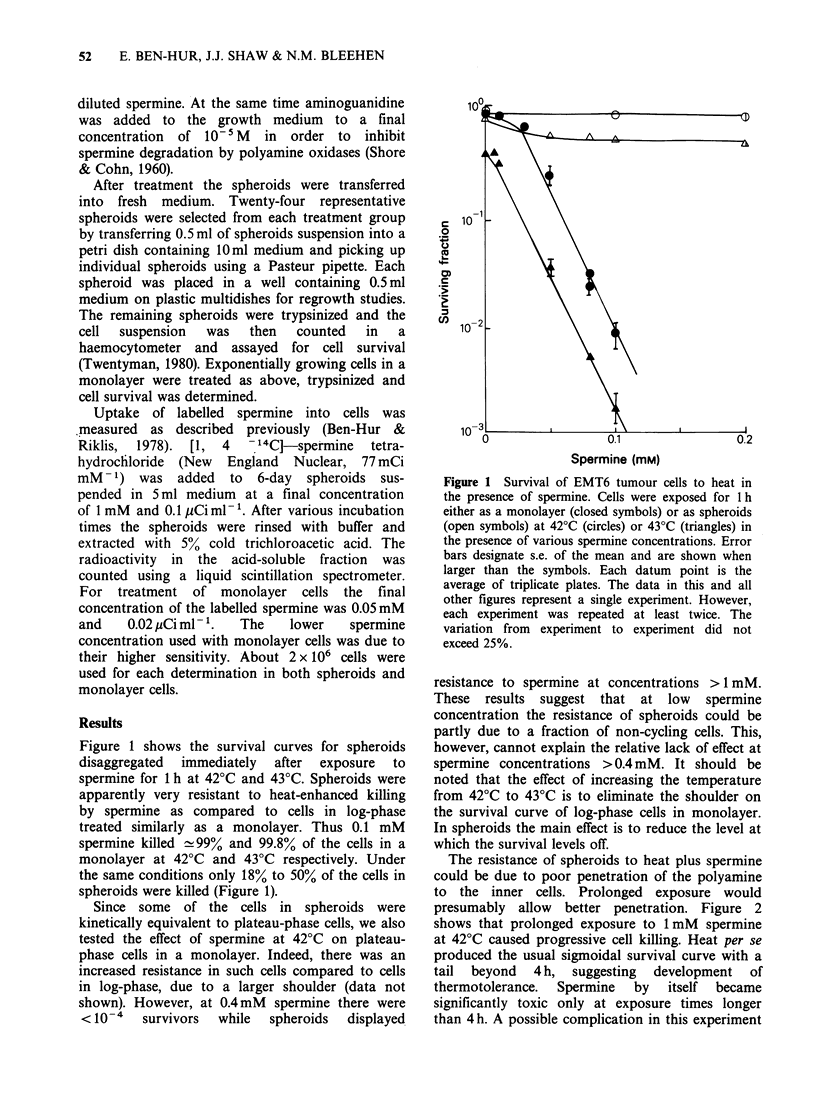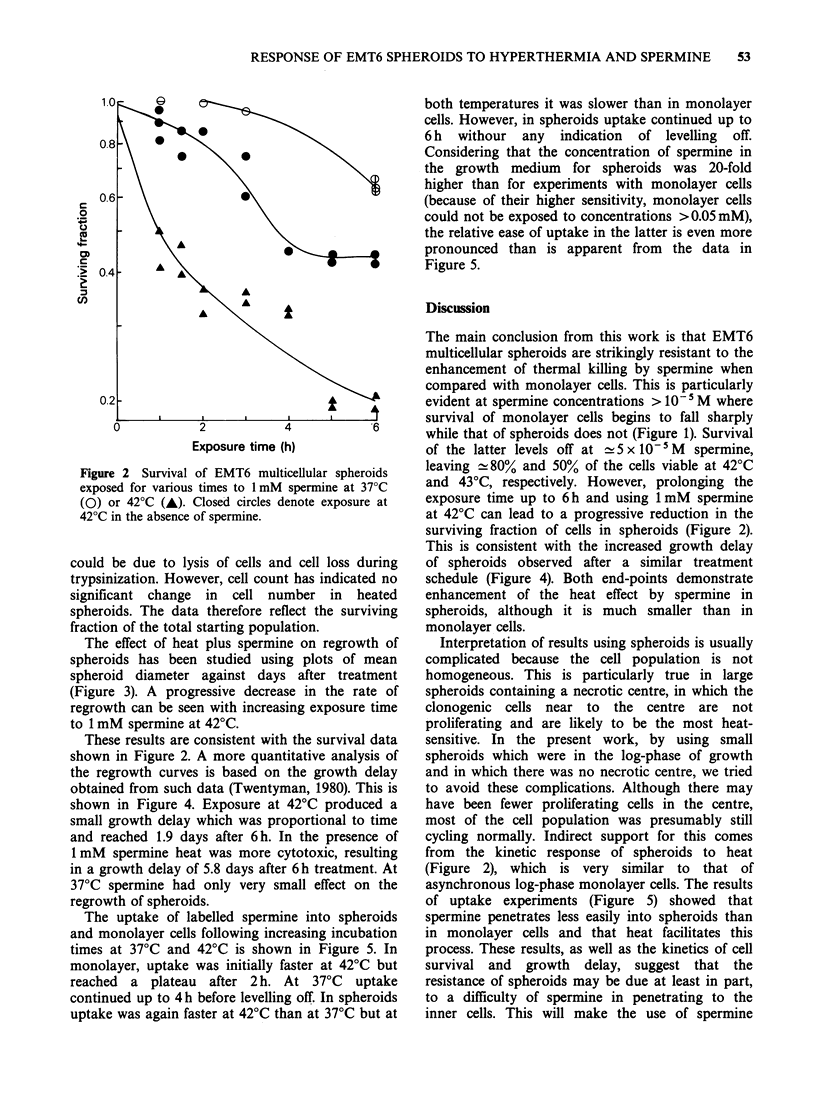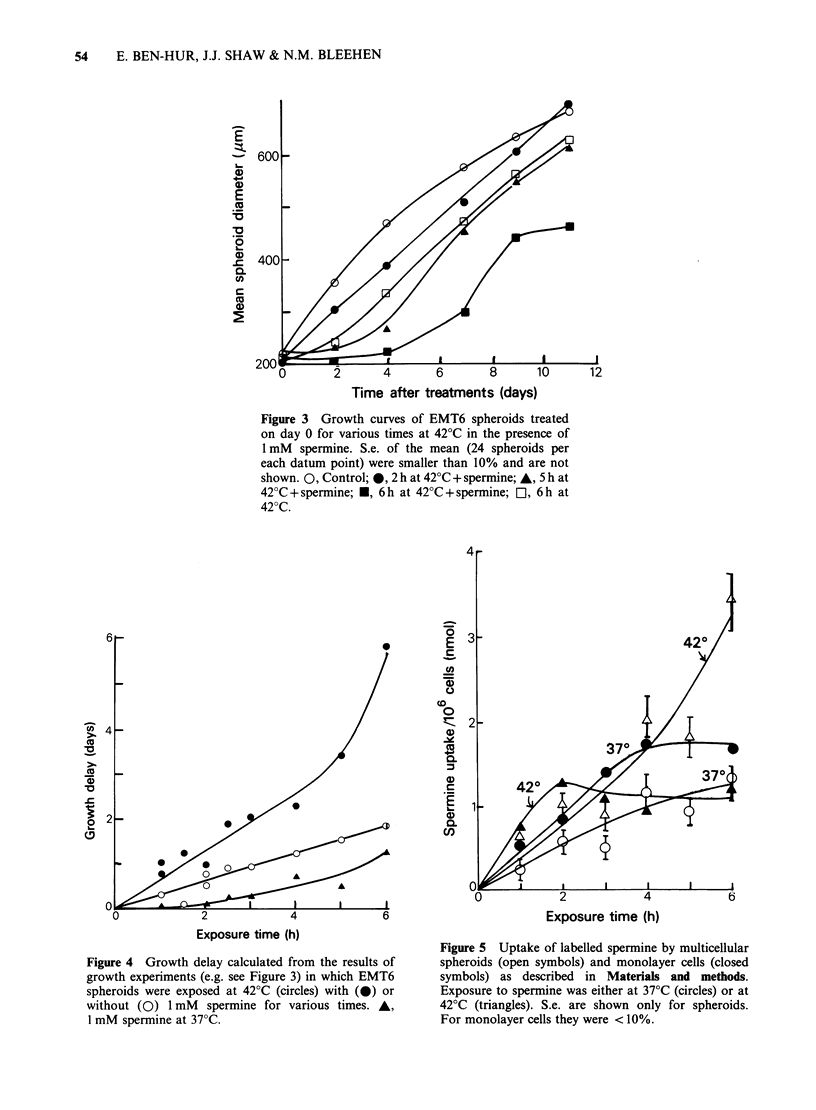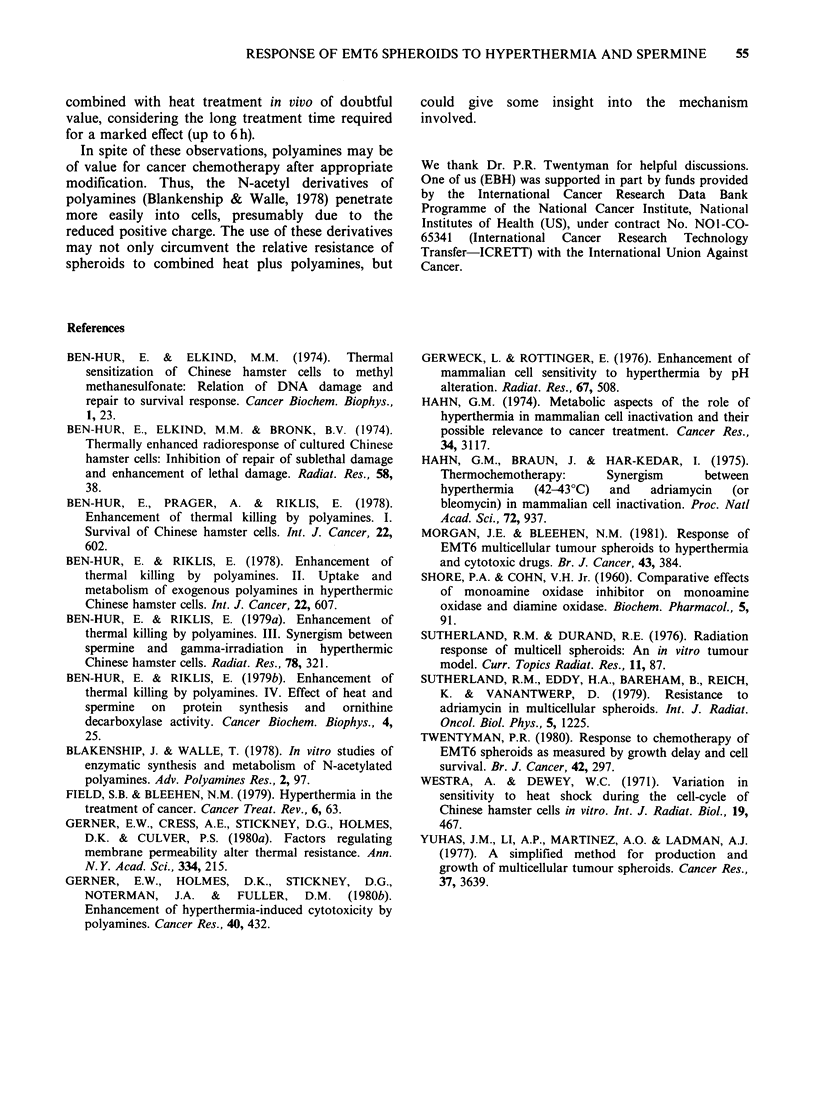# Enhancement of thermal killing by polyamines V. The response of EMT6 multicellular tumour spheroids versus monolayer cells.

**DOI:** 10.1038/bjc.1983.6

**Published:** 1983-01

**Authors:** E. Ben-Hur, J. J. Shaw, N. M. Bleehen

## Abstract

Polyamines, especially spermine, are very effective in enhancing thermal killing of mammalian cells cultured as a monolayer. The response of EMT6 multicellular tumour spheroids to heat in the presence of spermine was studied using cell survival and growth delay as endpoints. Compared to cells in a monolayer, spheroids were found to be highly resistant to combined heat and spermine. In spite of this, considerable enhancement of thermal killing by spermine was observed when the combined treatment was prolonged for a few hours. These results, together with data obtained using labelled spermine, suggest that difficulties in penetration of spermine into the inner cells of the spheroids contribute to the resistance of the latter. A method of circumventing this difficulty is discussed.


*Br. J. Cancer* (1983) **47**, 051–055

### Enhancement of thermal killing by polyamines V. The response of EMT6 multicellular tumour spheroids *versus* monolayer cells

E. Ben-Hur*, J.J. Shaw & N.M. Bleehen†

*University Department and MRC Unit of Clinical Oncology and Radiotherapeutics, The Medical School, Cambridge CB2 2QH.*

**Summary** Polyamines, especially spermine, are very effective in enhancing thermal killing of mammalian cells cultured as a monolayer. The response of EMT6 multicellular tumour spheroids to heat in the presence of spermine was studied using cell survival and growth delay as endpoints. Compared to cells in a monolayer, spheroids were found to be highly resistant to combined heat and spermine. In spite of this, considerable enhancement of thermal killing by spermine was observed when the combined treatment was prolonged for a few hours. These results, together with data obtained using labelled spermine, suggest that difficulties in penetration of spermine into the inner cells of the spheroids contribute to the resistance of the latter. A method of circumventing this difficulty is discussed.

Hyperthermia at temperatures above 41°C has recently gained increased interest as a potentially useful modality in cancer treatment (Field & Bleehen, 1979). Much of the rationale for considering the use of hyperthermia in cancer therapy has come from results obtained *in vitro.* Thus, hyperthermia sensitizes mammalian cells to radiation (Ben-Hur *et al.*, 1974) and drugs (Ben-Hur & Elkind, 1974; Hahn *et al.*, 1975) and preferentially kills cells in mid S-phase, which are normally more resistant to X-rays than in other stages of the cell cycle (Westra & Dewey, 1971). Thermal sensitivity is affected greatly by environmental factors. Nutritional deficiency (Hahn, 1974), lowered pH in the growth medium (Gerweck & Rottinger, 1976) and the naturally occurring polyamines when supplied exogenously (Ben-Hur *et al.*, 1978; Gerner *et al.*, 1980*a*), all enhance thermal response. The mechanism(s) leading to the enhanced response to polyamines are still obscure, although both the chromatin structure (Ben-Hur & Riklis, 1978; 1979*a,b*) and the plasma membrane (Gerner *et al.*, 1980*b*) have been implicated.

Multicellular tumour spheroids are an *in vitro* model system representing an intermediate level of complexity between monolayer cultures and solid tumours *in vivo* (Sutherland & Durand, 1976). This system has been used to study the response to hyperthermia and chemotherapy of various tumour cell lines. Thus, in EMT6 tumour spheroids, a marked resistance to adriamycin was shown when compared with exponentially growing monolayer cells (Sutherland *et al.*, 1979). We have described in a similar system a synergistic interaction between hyperthermia and the cytotoxic drugs bleomycin and adriamycin as measured by growth delay and cell survival (Morgan & Bleehen, 1981).

This paper demonstrates that, compared with monolayer cells, the EMT6 multicellular tumour spheroids are resistant to the combination of hyperthermia and spermine. The latter is the most effective polyamine in enhancing thermal response. Poor penetration of spermine to the inner cells of the spheroid appears to be part of the reason for this resistance.

### Materials and methods

The methods used for growth and assay of the EMT6/Ca/VJAC spheroid system were described previously (Twentyman, 1980; Morgan & Bleehen, 1981). Briefly, spheroids were grown from a single-cell suspension in culture flasks base coated with agar to prevent cell adhesion to the surface (Yuhas *et al.*, 1977). Spheroids were used in experiments on day 6, by which time their average diameter was $220 \pm 20\,\mu m$. Heat treatment was in a waterbath with the temperature controlled to $\pm 0.1$°C.

Spermine tetrahydrochloride was dissolved in water and stored at −20°C in small aliquots as a stock 0.1 M solution. Prior to use it was diluted in Hanks' balanced salt solution to a concentration 50-fold higher than the final concentration required in the growth medium. To each 4.9 ml spheroids suspension to be treated was added 0.1 ml of the

*Permanent address: Nuclear Research Center—Negev Dept. of Radiobiology, P.O. Box 9001, Beer-Sheva, Israel.
†Correspondence: N.M. Bleehen, Univ. Dept. & MRC Unit of Clinical Oncology and Radiotherapeutics, The Medical School, Cambridge CB2 2QH, UK.

Received 26 July 1982; accepted 23 September 1982.

0007-0920/83/010051–05 $01.00

© The Macmillan Press Ltd., 1983

diluted spermine. At the same time aminoguanidine was added to the growth medium to a final concentration of $10^{-5}$ M in order to inhibit spermine degradation by polyamine oxidases (Shore & Cohn, 1960).

After treatment the spheroids were transferred into fresh medium. Twenty-four representative spheroids were selected from each treatment group by transferring 0.5 ml of spheroids suspension into a petri dish containing 10 ml medium and picking up individual spheroids using a Pasteur pipette. Each spheroid was placed in a well containing 0.5 ml medium on plastic multidishes for regrowth studies. The remaining spheroids were trypsinized and the cell suspension was then counted in a haemocytometer and assayed for cell survival (Twentyman, 1980). Exponentially growing cells in a monolayer were treated as above, trypsinized and cell survival was determined.

Uptake of labelled spermine into cells was measured as described previously (Ben-Hur & Riklis, 1978). [1, 4 $^{-14}$C]—spermine tetrahydrochloride (New England Nuclear, 77 mCi $mM^{-1}$) was added to 6-day spheroids suspended in 5 ml medium at a final concentration of 1 mM and 0.1 $\mu Ci\,ml^{-1}$. After various incubation times the spheroids were rinsed with buffer and extracted with 5% cold trichloroacetic acid. The radioactivity in the acid-soluble fraction was counted using a liquid scintillation spectrometer. For treatment of monolayer cells the final concentration of the labelled spermine was 0.05 mM and 0.02 $\mu Ci\,ml^{-1}$. The lower spermine concentration used with monolayer cells was due to their higher sensitivity. About $2\times10^6$ cells were used for each determination in both spheroids and monolayer cells.

### Results

Figure 1 shows the survival curves for spheroids disaggregated immediately after exposure to spermine for 1 h at 42°C and 43°C. Spheroids were apparently very resistant to heat-enhanced killing by spermine as compared to cells in log-phase treated similarly as a monolayer. Thus 0.1 mM spermine killed $\simeq$99% and 99.8% of the cells in a monolayer at 42°C and 43°C respectively. Under the same conditions only 18% to 50% of the cells in spheroids were killed (Figure 1).

Since some of the cells in spheroids were kinetically equivalent to plateau-phase cells, we also tested the effect of spermine at 42°C on plateau-phase cells in a monolayer. Indeed, there was an increased resistance in such cells compared to cells in log-phase, due to a larger shoulder (data not shown). However, at 0.4 mM spermine there were $<10^{-4}$ survivors while spheroids displayed resistance to spermine at concentrations >1 mM. These results suggest that at low spermine concentration the resistance of spheroids could be partly due to a fraction of non-cycling cells. This, however, cannot explain the relative lack of effect at spermine concentrations >0.4 mM. It should be noted that the effect of increasing the temperature from 42°C to 43°C is to eliminate the shoulder on the survival curve of log-phase cells in monolayer. In spheroids the main effect is to reduce the level at which the survival levels off.

**Figure 1** Survival of EMT6 tumour cells to heat in the presence of spermine. Cells were exposed for 1 h either as a monolayer (closed symbols) or as spheroids (open symbols) at 42°C (circles) or 43°C (triangles) in the presence of various spermine concentrations. Error bars designate s.e. of the mean and are shown when larger than the symbols. Each datum point is the average of triplicate plates. The data in this and all other figures represent a single experiment. However, each experiment was repeated at least twice. The variation from experiment to experiment did not exceed 25%.

The resistance of spheroids to heat plus spermine could be due to poor penetration of the polyamine to the inner cells. Prolonged exposure would presumably allow better penetration. Figure 2 shows that prolonged exposure to 1 mM spermine at 42°C caused progressive cell killing. Heat *per se* produced the usual sigmoidal survival curve with a tail beyond 4 h, suggesting development of thermotolerance. Spermine by itself became significantly toxic only at exposure times longer than 4 h. A possible complication in this experiment

**Figure 2** Survival of EMT6 multicellular spheroids exposed for various times to 1 mM spermine at 37°C (○) or 42°C (▲). Closed circles denote exposure at 42°C in the absence of spermine.

could be due to lysis of cells and cell loss during trypsinization. However, cell count has indicated no significant change in cell number in heated spheroids. The data therefore reflect the surviving fraction of the total starting population.

The effect of heat plus spermine on regrowth of spheroids has been studied using plots of mean spheroid diameter against days after treatment (Figure 3). A progressive decrease in the rate of regrowth can be seen with increasing exposure time to 1 mM spermine at 42°C.

These results are consistent with the survival data shown in Figure 2. A more quantitative analysis of the regrowth curves is based on the growth delay obtained from such data (Twentyman, 1980). This is shown in Figure 4. Exposure at 42°C produced a small growth delay which was proportional to time and reached 1.9 days after 6 h. In the presence of 1 mM spermine heat was more cytotoxic, resulting in a growth delay of 5.8 days after 6 h treatment. At 37°C spermine had only very small effect on the regrowth of spheroids.

The uptake of labelled spermine into spheroids and monolayer cells following increasing incubation times at 37°C and 42°C is shown in Figure 5. In monolayer, uptake was initially faster at 42°C but reached a plateau after 2 h. At 37°C uptake continued up to 4 h before levelling off. In spheroids uptake was again faster at 42°C than at 37°C but at both temperatures it was slower than in monolayer cells. However, in spheroids uptake continued up to 6 h withour any indication of levelling off. Considering that the concentration of spermine in the growth medium for spheroids was 20-fold higher than for experiments with monolayer cells (because of their higher sensitivity, monolayer cells could not be exposed to concentrations >0.05 mM), the relative ease of uptake in the latter is even more pronounced than is apparent from the data in Figure 5.

### Discussion

The main conclusion from this work is that EMT6 multicellular spheroids are strikingly resistant to the enhancement of thermal killing by spermine when compared with monolayer cells. This is particularly evident at spermine concentrations $>10^{-5}$ M where survival of monolayer cells begins to fall sharply while that of spheroids does not (Figure 1). Survival of the latter levels off at $\simeq 5\times10^{-5}$ M spermine, leaving $\simeq$80% and 50% of the cells viable at 42°C and 43°C, respectively. However, prolonging the exposure time up to 6 h and using 1 mM spermine at 42°C can lead to a progressive reduction in the surviving fraction of cells in spheroids (Figure 2). This is consistent with the increased growth delay of spheroids observed after a similar treatment schedule (Figure 4). Both end-points demonstrate enhancement of the heat effect by spermine in spheroids, although it is much smaller than in monolayer cells.

Interpretation of results using spheroids is usually complicated because the cell population is not homogeneous. This is particularly true in large spheroids containing a necrotic centre, in which the clonogenic cells near to the centre are not proliferating and are likely to be the most heat-sensitive. In the present work, by using small spheroids which were in the log-phase of growth and in which there was no necrotic centre, we tried to avoid these complications. Although there may have been fewer proliferating cells in the centre, most of the cell population was presumably still cycling normally. Indirect support for this comes from the kinetic response of spheroids to heat (Figure 2), which is very similar to that of asynchronous log-phase monolayer cells. The results of uptake experiments (Figure 5) showed that spermine penetrates less easily into spheroids than in monolayer cells and that heat facilitates this process. These results, as well as the kinetics of cell survival and growth delay, suggest that the resistance of spheroids may be due at least in part, to a difficulty of spermine in penetrating to the inner cells. This will make the use of spermine

**Figure 3** Growth curves of EMT6 spheroids treated on day 0 for various times at 42°C in the presence of 1 mM spermine. S.e. of the mean (24 spheroids per each datum point) were smaller than 10% and are not shown. ○, Control; ●, 2 h at 42°C + spermine; ▲, 5 h at 42°C + spermine; ■, 6 h at 42°C + spermine; □, 6 h at 42°C.

**Figure 4** Growth delay calculated from the results of growth experiments (e.g. see Figure 3) in which EMT6 spheroids were exposed at 42°C (circles) with (●) or without (○) 1 mM spermine for various times. ▲, 1 mM spermine at 37°C.

**Figure 5** Uptake of labelled spermine by multicellular spheroids (open symbols) and monolayer cells (closed symbols) as described in **Materials and methods**. Exposure to spermine was either at 37°C (circles) or at 42°C (triangles). S.e. are shown only for spheroids. For monolayer cells they were < 10%.

combined with heat treatment *in vivo* of doubtful value, considering the long treatment time required for a marked effect (up to 6 h).

In spite of these observations, polyamines may be of value for cancer chemotherapy after appropriate modification. Thus, the N-acetyl derivatives of polyamines (Blankenship & Walle, 1978) penetrate more easily into cells, presumably due to the reduced positive charge. The use of these derivatives may not only circumvent the relative resistance of spheroids to combined heat plus polyamines, but could give some insight into the mechanism involved.

We thank Dr. P.R. Twentyman for helpful discussions. One of us (EBH) was supported in part by funds provided by the International Cancer Research Data Bank Programme of the National Cancer Institute, National Institutes of Health (US), under contract No. NO1-CO-65341 (International Cancer Research Technology Transfer—ICRETT) with the International Union Against Cancer.

**References**

BEN-HUR, E. & ELKIND, M.M. (1974). Thermal sensitization of Chinese hamster cells to methyl methanesulfonate: Relation of DNA damage and repair to survival response. *Cancer Biochem. Biophys.*, **1,** 23.

BEN-HUR, E., ELKIND, M.M. & BRONK, B.V. (1974). Thermally enhanced radioresponse of cultured Chinese hamster cells: Inhibition of repair of sublethal damage and enhancement of lethal damage. *Radiat. Res.*, **58,** 38.

BEN-HUR, E., PRAGER, A. & RIKLIS, E. (1978). Enhancement of thermal killing by polyamines. I. Survival of Chinese hamster cells. *Int. J. Cancer*, **22,** 602.

BEN-HUR, E. & RIKLIS, E. (1978). Enhancement of thermal killing by polyamines. II. Uptake and metabolism of exogenous polyamines in hyperthermic Chinese hamster cells. *Int. J. Cancer*, **22,** 607.

BEN-HUR, E. & RIKLIS, E. (1979*a*). Enhancement of thermal killing by polyamines. III. Synergism between spermine and gamma-irradiation in hyperthermic Chinese hamster cells. *Radiat. Res.*, **78,** 321.

BEN-HUR, E. & RIKLIS, E. (1979*b*). Enhancement of thermal killing by polyamines. IV. Effect of heat and spermine on protein synthesis and ornithine decarboxylase activity. *Cancer Biochem. Biophys.*, **4,** 25.

BLAKENSHIP, J. & WALLE, T. (1978). *In vitro* studies of enzymatic synthesis and metabolism of N-acetylated polyamines. *Adv. Polyamines Res.*, **2,** 97.

FIELD, S.B. & BLEEHEN, N.M. (1979). Hyperthermia in the treatment of cancer. *Cancer Treat. Rev.*, **6,** 63.

GERNER, E.W., CRESS, A.E., STICKNEY, D.G., HOLMES, D.K. & CULVER, P.S. (1980*a*). Factors regulating membrane permeability alter thermal resistance. *Ann. N.Y. Acad. Sci.*, **334,** 215.

GERNER, E.W., HOLMES, D.K., STICKNEY, D.G., NOTERMAN, J.A. & FULLER, D.M. (1980*b*). Enhancement of hyperthermia-induced cytotoxicity by polyamines. *Cancer Res.*, **40,** 432.

GERWECK, L. & ROTTINGER, E. (1976). Enhancement of mammalian cell sensitivity to hyperthermia by pH alteration. *Radiat. Res.*, **67,** 508.

HAHN, G.M. (1974). Metabolic aspects of the role of hyperthermia in mammalian cell inactivation and their possible relevance to cancer treatment. *Cancer Res.*, **34,** 3117.

HAHN, G.M., BRAUN, J. & HAR-KEDAR, I. (1975). Thermochemotherapy: Synergism between hyperthermia (42–43°C) and adriamycin (or bleomycin) in mammalian cell inactivation. *Proc. Natl Acad. Sci.*, **72,** 937.

MORGAN, J.E. & BLEEHEN, N.M. (1981). Response of EMT6 multicellular tumour spheroids to hyperthermia and cytotoxic drugs. *Br. J. Cancer*, **43,** 384.

SHORE, P.A. & COHN, V.H. Jr. (1960). Comparative effects of monoamine oxidase inhibitor on monoamine oxidase and diamine oxidase. *Biochem. Pharmacol.*, **5,** 91.

SUTHERLAND, R.M. & DURAND, R.E. (1976). Radiation response of multicell spheroids: An *in vitro* tumour model. *Curr. Topics Radiat. Res.*, **11,** 87.

SUTHERLAND, R.M., EDDY, H.A., BAREHAM, B., REICH, K. & VANANTWERP, D. (1979). Resistance to adriamycin in multicellular spheroids. *Int. J. Radiat. Oncol. Biol. Phys.*, **5,** 1225.

TWENTYMAN, P.R. (1980). Response to chemotherapy of EMT6 spheroids as measured by growth delay and cell survival. *Br. J. Cancer*, **42,** 297.

WESTRA, A. & DEWEY, W.C. (1971). Variation in sensitivity to heat shock during the cell-cycle of Chinese hamster cells *in vitro*. *Int. J. Radiat. Biol.*, **19,** 467.

YUHAS, J.M., LI, A.P., MARTINEZ, A.O. & LADMAN, A.J. (1977). A simplified method for production and growth of multicellular tumour spheroids. *Cancer Res.*, **37,** 3639.